# Azacitidine-Induced Pneumonitis in a Patient With Acute Myeloid Leukemia and Hyperleukocytosis

**DOI:** 10.7759/cureus.26758

**Published:** 2022-07-11

**Authors:** Rafaella Litvin, Mona Dasgupta, Mohamed Saad Eldin, Mihir Shah, Sherene Fakhran

**Affiliations:** 1 Department of Internal Medicine, John H. Stroger, Jr. Hospital of Cook County, Chicago, USA; 2 Department of Pulmonary, Critical Care and Sleep Medicine, John H. Stroger, Jr. Hospital of Cook County, Chicago, USA

**Keywords:** acute hypoxemic respiratory failure, chemotherapy-related toxicity, drug induced pneumonitis, acute myeloid leukemia (aml), azacitidine, pulmonary toxicity

## Abstract

Chemotherapy-related toxicity is a complex aspect of oncologic care. Pulmonary toxicity, in particular, poses a significant challenge, as it can have diverse presentations and can closely mimic other common complications of cancer treatment, such as infections. Azacitidine is an agent widely employed in high-risk myelodysplastic syndrome and acute myeloid leukemia. We present a case of azacitidine-induced pneumonitis, a rare adverse effect, in a 70-year-old patient with acute myeloid leukemia (AML) and hyperleukocytosis. After discontinuation of the drug and introduction of steroids, the patient had complete resolution of symptoms, highlighting the importance of identifying and addressing chemotherapy-induced pneumonitis.

## Introduction

Chemotherapy-induced pulmonary toxicity is an often unrecognized cause of respiratory failure in oncologic patients. It can be challenging to diagnose, as the presentation can closely resemble infections, malignant infiltration, and cardiogenic pulmonary edema, and there are no known specific findings on exam or imaging [1]. 

Azacitidine is a hypomethylating agent which has become the standard of care for patients with high-risk myelodysplastic syndrome or acute myeloid leukemia (AML) who are not eligible for high-intensity chemotherapy [2,3]. It is known to cause pancytopenia and gastrointestinal side effects but has seldom been reported to lead to pulmonary complications.

Here we present a case of azacitidine-induced pulmonary toxicity, a rare adverse effect, in a 70-year-old patient with AML and hyperleukocytosis. Our patient developed hypoxic respiratory failure on day four of his second cycle of azacitidine. The presentation was initially thought to be infectious in nature; however, the patient continued to worsen while on broad-spectrum antibiotics, and the infectious workup was unrevealing. Azacitidine-induced pulmonary toxicity was suspected, and he was started on steroids, with complete resolution of respiratory symptoms.

## Case presentation

A 70-year-old male with a history of acute myeloid leukemia (AML), chronic obstructive pulmonary disease (COPD), lipoid pneumonia related to cocaine use, peripheral vascular disease, latent tuberculosis, and sickle cell trait was admitted to the hospital for one week of recurrent falls at home, and worsening residual left lower extremity weakness. 

He had been diagnosed with AML ten months prior but was deemed unfit for intense induction chemotherapy due to multiple comorbidities. He received one cycle of azacitidine and venetoclax with no respiratory complications. The patient declined further treatment due to side effects of progressive cerebrovascular events, including slurred speech and left-sided weakness, two months after the initial diagnosis.

**Figure 1:**
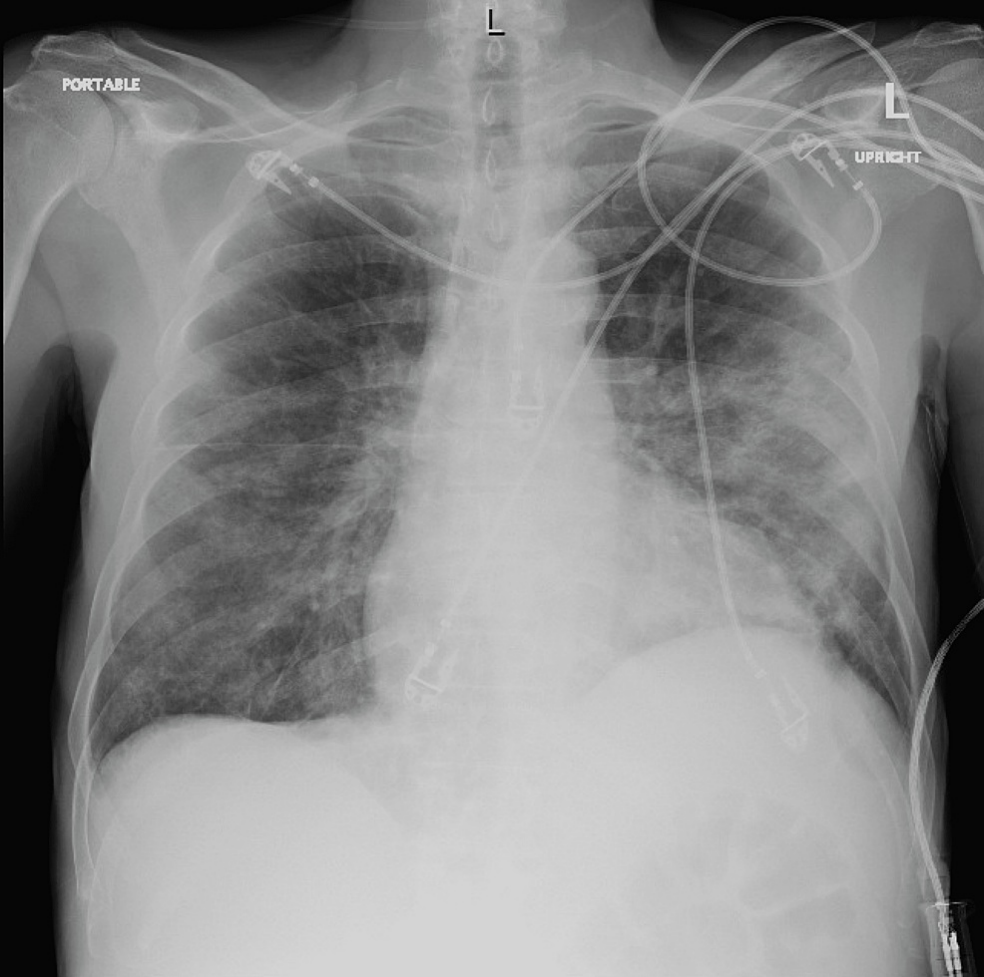
Chest X-ray on the day of admission

Notable laboratory values on admission are shown in Table 1. Oxygen saturation was 98% on room air. Chest X-ray at the time (Figure 1) showed a small left pneumothorax, and residual pulmonary opacities thought to be related to his history of lipoid pneumonia. The patient was admitted to the intensive care unit (ICU) for hyperleukocytosis, acute kidney injury, and tumor lysis syndrome.

**Table 1:** Laboratory values on admission

Test	Value on admission	Reference values
Hemoglobin	4.3 g/dL	12.9 - 16.8 g/dL
White blood cells	108.2 k/μL	4.4 - 10.6 k/μL
Percentage of blasts	88%	0%
Platelets	29 k/μL	161 - 369 k/μL
Creatinine	1.9 mg/dL	0.6 - 1.4 mg/dL
Blood urea nitrogen	26 mg/dL	8 - 20 mg/dL

**Figure 2:**
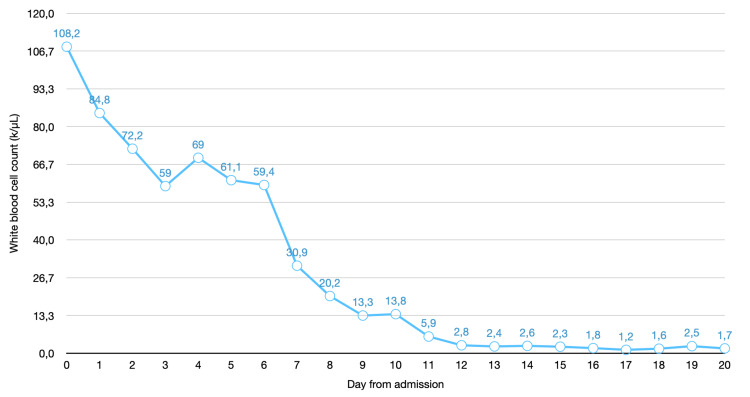
White blood count trend during admission The Y-axis represents white blood count in k/μL, the X-axis represents the day from admission. Hydroxyurea was started on day one, azacitidine was started on day two.

It was thought that the patient's presentation of increased weakness and falls was related to hyperleukocytosis. Therefore, on day two of hospital admission, he was started on azacitidine with hydroxyurea for cytoreduction of AML with hyperleukocytosis, with the intention to treat for seven days. Over the course of admission, the white blood cell count decreased rapidly (Figure 2). He developed a temperature of 39°C in the evening after initiation of azacitidine. The patient was started on empiric antibiotics, including levofloxacin, followed by cefepime, and an initial infectious workup was ordered, including blood and sputum cultures. 

**Figure 3:**
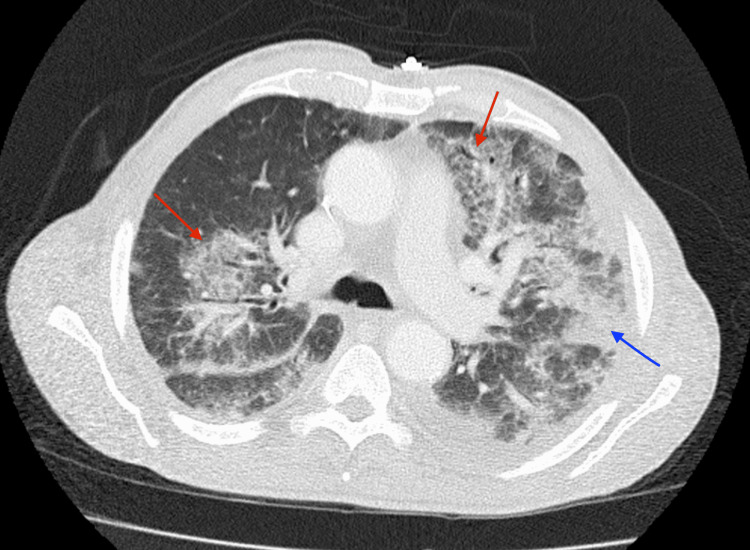
Computed tomography of the chest on day four of azacitidine Red arrows indicate ground-glass opacities, blue arrow indicates alveolar opacities.

Over the course of the next two days, the patient continued to have intermittent fevers with temperatures up to 39°C. On the evening after day four of azacitidine treatment, he developed tachycardia, tachypnea, and hypoxemia. While on six liters of oxygen through a nasal cannula, the patient's saturation remained between 85-92%. He was started on high-flow nasal oxygen of 60 liters with a fraction of inspired oxygen (FiO_2_) of 50%. Antibiotic coverage was broadened to piperacillin-tazobactam. Azacitidine was held due to concern for infection, with the patient having completed four of seven days of treatment. A full infectious workup was initiated, and the patient was pan-cultured as well as pan-scanned. Computed tomography of the chest (Figure 3) showed bilateral pleural effusions with associated atelectasis, patchy bilateral ground glass, and airspace opacities which were concerning for possible pneumonia.

**Figure 4:**
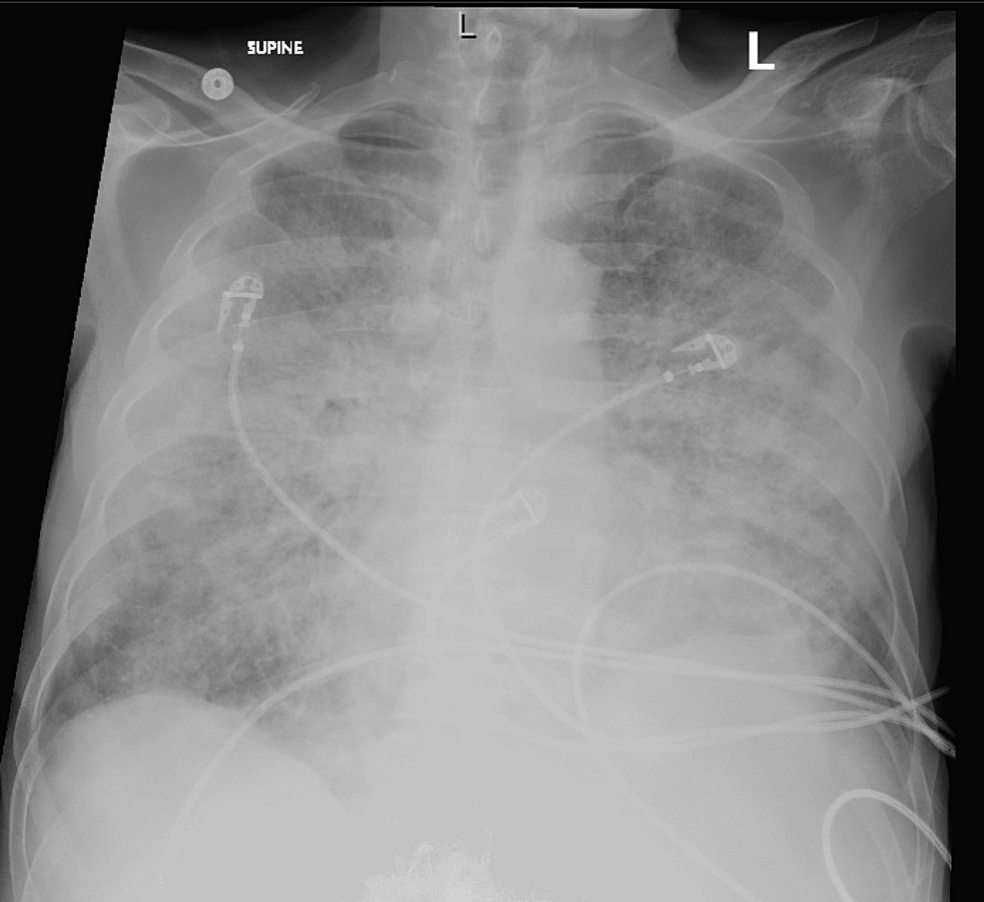
Chest X-ray four days after the beginning of symptoms and discontinuation of azacitidine

Over the following four days, the patient's oxygenation continued to worsen despite treatment for possible infections, and his chest X-ray (Figure 4) showed worsening bilateral pulmonary opacities. He was started on methylprednisolone at a dose of 1 mg/kg due to concern for pneumonitis, and antibiotic coverage was again broadened to vancomycin and meropenem, of which he would complete a 10-day and 11-day course, respectively.

Four days later, and eight days after azacitidine was discontinued, the patient was transitioned to room air, with an oxygen saturation of 97%. He was transferred from the medical ICU to general medicine floors for further management.

Differential diagnosis

The differential diagnosis for acute hypoxic respiratory failure in our patient included bacterial, viral, or fungal pneumonia, leukemic infiltration, pulmonary embolism, worsening of known previous lipoid pneumonia, and chemotherapy-related pneumonitis. Worsening of his lipoid pneumonia was low on the differential as the patient had not used cocaine or other drugs prior to admission. Additionally, leukemic infiltration was unlikely, as azacitidine led to a marked improvement of his blood counts.

The following infectious workup was completed on the patient: blood cultures (a total of five sets), sputum culture, urine culture (a total of two), legionella antigen, histoplasma antigen, blastomycosis antigen, beta-D glucan, methicillin-resistant S. aureus (MRSA) nares, influenza viral panel, respiratory viral panel, and two COVID-19 swabs. The final results were negative for all of the studies performed.

Bronchoscopy with bronchoalveolar was not performed, initially due to patient preference and later due to the marked improvement of respiratory symptoms with the initiation of steroids. 

Treatment

Upon initiating treatment with azacitidine, the patient received cytoreduction therapy with hydroxyurea, and prophylactic voriconazole, acyclovir, and trimethoprim-sulfamethoxazole. He then received four out of seven planned days of 130 mg of azacitidine before the medication was stopped due to worsening acute hypoxic respiratory failure. Due to concern for infection, the patient completed a course of broad-spectrum antibiotics, including (sequentially): levofloxacin, azithromycin, cefepime, piperacillin-tazobactam, vancomycin, and meropenem, with no improvement. The patient was started on a 12-day steroid taper with methylprednisolone 60 mg (1 mg/kg) four days after he developed respiratory complications, after which he soon had symptom resolution. 

The patient's non-pharmacological treatment centered around targeting his hypoxemia. He initially required oxygen supplementation via a high-flow nasal cannula. He was later weaned to a nasal cannula and finally to room air.

Outcome and follow-up 

Within five days of starting steroids, respiratory symptoms improved, and the patient went from requiring oxygen supplementation with a high-flow nasal cannula to saturating at 95% on room air.

He was transitioned from the intensive care unit to general medicine, where he experienced persistent left lower extremity pain due to peripheral artery disease with occlusion of vascular graft and dry gangrene. After partial recovery from chemotherapy-induced pancytopenia, the patient underwent a left below-knee amputation. 

The plan was to restart chemotherapy after the surgical procedure. However, the patient and his family repeatedly declined any further cancer-directed therapy during his hospital admission, and he was discharged to a skilled nursing facility one month after the initial presentation. 

On follow-up, one month after discharge, the patient continued to decline any leukemia-directed therapy. He was asymptomatic from a respiratory standpoint and was recovering adequately from the lower extremity amputation.

## Discussion

Acute respiratory failure is a common complication in patients undergoing chemotherapy for acute leukemia, and it is a major cause of mortality. The initial concern in most cases is respiratory failure secondary to an infectious cause, and all patients are empirically treated. However, cultures are frequently negative [4]. 

If patients do not improve on appropriate antibiotics and the infectious work-up is noncontributory, it is important to consider other etiologies for respiratory failure. For example, pulmonary leukemic infiltration has been found to occur almost as frequently as pneumonia in this population on post-mortem examination [5]. Additionally, exacerbations of chronic lung disease, pulmonary edema, and drug toxicity can present in a similar fashion and are important differential diagnoses. Of these, chemotherapy-related adverse events can easily be overlooked, especially when patients are not receiving agents known to classically lead to pulmonary toxicity, such as bleomycin, methotrexate, and cyclophosphamide [1].

Azacitidine is a hypomethylating agent, which has become the standard of care for patients with high-risk myelodysplastic syndrome or AML who are not eligible for high-intensity chemotherapy [2,3]. The most common side effects of hypomethylating agents include pancytopenia, abdominal pain, constipation, ecchymoses, and infections. 

Azacitidine-induced pulmonary toxicity is an extremely rare adverse effect, with less than twenty cases reported worldwide. It is described as interstitial pneumonitis, and clinical manifestations include fever, dyspnea, cough, and hypoxemia. The most common radiologic findings are interstitial or ground-glass opacities, with some cases also reporting consolidation and airway opacities [6-9]. Bronchoscopy with bronchoalveolar lavage (BAL) can aid in excluding alternative diagnosis, and biopsy may show patterns characteristic of drug-related toxicity, such as organizing pneumonia [10]. The timing from drug administration and symptom onset is highly variable (days to weeks). Furthermore, toxicity has been reported in patients who previously tolerated cycles of azacitidine with no complications, such as our patient. 

The management generally involves discontinuing the drug and initiating steroids (dose and duration of treatment are variable). Patients tend to improve significantly after steroid treatment [11-13]. In the reported cases in which patients were later rechallenged with azacitidine, they then presented with recurrent, more severe respiratory symptoms [10,13-14].

## Conclusions

Chemotherapy-induced pneumonitis should be considered in leukemia and myelodysplastic syndrome (MDS) patients with acute respiratory decompensation who do not improve with antibiotics, even when the receiving chemotherapy agent is not commonly known to cause pulmonary toxicity. Azacitidine-induced pneumonitis is a rare adverse reaction, estimated to occur in less than 0.1% of patients. Most cases improve with discontinuation of the drug and intravenous or oral steroids. Pulmonary toxicity can occur even in patients previously treated with azacitidine without complications. If a patient develops pulmonary toxicity, recurrence upon repeated exposure is likely, and alternative therapies should be considered.
